# Biofilm-Formation Ability and the Presence of Adhesion Genes in Coagulase-Negative Staphylococci Isolates from Chicken Broilers

**DOI:** 10.3390/ani11030728

**Published:** 2021-03-07

**Authors:** Agnieszka Marek, Ewelina Pyzik, Dagmara Stępień-Pyśniak, Marta Dec, Łukasz S. Jarosz, Anna Nowaczek, Magdalena Sulikowska

**Affiliations:** 1 Sub-Department of Preventive Veterinary and Avian Diseases, Faculty of Veterinary Medicine, Institute of Biological Bases of Animal Diseases, University of Life Sciences in Lublin, 20-950 Lublin, Poland; ewelina.pyzik@up.lublin.pl (E.P.); dagmara.stepien@up.lublin.pl (D.S.-P.); marta.dec@up.lublin.pl (M.D.); anna.nowaczek@up.lublin.pl (A.N.); 2 Department of Epizootiology and Clinic of Infectious Diseases, Faculty of Veterinary Medicine, University of Life Sciences in Lublin, Głęboka 30, 20-612 Lublin, Poland; lukasz.jarosz@up.lublin.pl; 3 Eskulap-Veterinary Clinic, 37-310 Nowa Sarzyna, Poland; magtom@go2.pl

**Keywords:** biofilm, biofilm-associated genes, coagulase-negative staphylococci, broiler chickens

## Abstract

**Simple Summary:**

Bacteria of the genus *Staphylococcus* are universally present on the mucous membranes and skin of warm-blooded animals. They are divided into two groups on the basis of their ability to clot blood plasma: the coagulase-positive (CoPS) and coagulase-negative staphylococci (CoNS). Some species can cause opportunistic infections in poultry. Identification and characterization of strains of the genus *Staphylococcus* isolated from farm animals are crucial in epidemiological research and for developing effective methods to treat infections and food poisoning induced by these bacteria. The main virulence factor of coagulase-negative staphylococci is considered to be their ability to form complex biofilm structures on the surfaces of damaged tissues. Biofilms increase the invasive properties of CoNS and their ability to cause infection. The purpose of this study was to determine the biofilm-forming potential of coagulase-negative *Staphylococcus* strains isolated from poultry. The frequency of selected genes potentially playing a role in the biofilm formation process was also determined. The results of the study indicate that the majority (79.3%) of CoNS isolated from broiler chickens in this study were capable of producing a biofilm.

**Abstract:**

The aim of the study was to analyze the biofilm-production capacity of 87 coagulase-negative *Staphylococcus* strains (CoNS) isolated from broiler chickens and to determine the occurrence of biofilm-associated genes. The biofilm production capacity of staphylococci was assessed using the microtiter plate method (MTP), and the frequency of genes was determined by PCR. The ability to form a biofilm in vitro was shown in 79.3% of examined strains. Strong biofilm capacity was demonstrated in 26.4% of strains, moderate capacity in 25.3%, weak capacity in 27.6%, and a complete lack of biofilm production capacity in 20.7% of strains. The *ica*AB gene responsible for the production of extracellular polysaccharide adhesins was detected in 6.9% of strains. The other four genes, i.e., *bap* (encoding biofilm-associated protein), *atl*E (encoding cell surface protein exhibiting vitronectin-binding activity), *fbe* (encoding fibrinogen-binding protein), and *eno* (encoding laminin-binding protein) were detected in 5.7%, 19.5%, 8%, and 70.1% of strains, respectively. Demonstration of genes that play a role in bacterial biofilm formation may serve as a genetic basis to distinguish between symbiotic and potentially invasive coagulase-negative staphylococcal strains.

## 1. Introduction

Bacteria of the genus *Staphylococcus* are ubiquitous in the environment, including the water, soil, and air, and are isolated from various animal species, including poultry. The genus *Staphylococcus* currently includes almost 60 species, and within some of these species, subspecies have been distinguished [1]. Traditionally, they are divided into two groups on the basis of their ability to clot blood plasma (the coagulase reaction). From a clinical perspective, particularly harmful species include coagulase-positive staphylococci, such as *S. aureus, S. intermedius, S. schleiferi* subsp. *coagulans, S. pseudointermedius, S. lutrae,* and *S. delphini*, and some strains of the species *S. hyicus*. The most important of these is *S. aureus*, which, apart from organ infections, can produce as many as 25 different toxins causing severe food poisoning [2]. The remaining *Staphylococcus* species, which are coagulase-negative (CoNS), usually induce infections in individuals with reduced immunity. CoNS are natural in the microbiota of humans and animals. They inhabit the digestive tract and respiratory system and are present as physiological biota on the skin and mucous membranes of humans and animals. CoNS have been isolated from clinically infected chickens with cellulitis, granulomas in the liver and lungs, and gangrenous dermatitis or subcutaneous abscesses. *S. xylosus* and *S. simulans* have been recovered from infected bones and in the course of endocarditis [3,4,5]. These staphylococcal species can also induce subclinical disease with histopathological lesions in the liver, spleen, and intestines of infected chickens [6,7]. Compared to *S. aureus*, CoNS do not produce a large number of toxic enzymes and toxins [1]. Factors affecting their virulence include structures that compose the bacterial cell, as well as substances and structures produced extracellularly but integrally associated with the cell, e.g., mucus or adhesins [8]. These microorganisms were long-considered non-pathogenic, and isolation from material collected from sick individuals was treated as contamination [9]. However, due to their implication in infections in both humans and animals, research interest in CoNS has increased over the past decade [10,11,12]. Many species, such as *S. gallinarum*, *S. arlettae*, *S. chromogenes*, *S. xylosus*, and *S. epidermidis*, have commonly been isolated from the nares and skin of healthy chickens, but some of them have also been isolated from cases of dermatitis tendinitis and endocarditis in chickens [3,5,13,14,15]. Moreover, chicken meat products are susceptible to contamination by these bacteria during post-slaughter processing. The contamination is mainly attributed to poor carcasses handling or cross-contamination during meat processing [7]. In recent years, species such as *S. equorum, S. saprophyticus, S. haemolyticus, S. xylosus*, and especially *S. epidermidis* have also been isolated from clinical specimens collected from humans [9,16]. Among coagulase-negative staphylococci, *S. epidermidis* and *S. saprophyticus* have the greatest pathogenic potential and diversity. *S. epidermidis* strains from different sources exhibit genetic differences. For example, *S. epidermidis* isolated from infections associated with clinical catheters has been reported to differ from those isolated from other environments [17,18,19]. Different origins of isolates, e.g., from animals or air, may affect their adherence and/or capability of forming a biofilm

The main virulence factor in this group of bacteria is considered to be their ability to form complex biofilm structures on the surfaces of damaged tissues [20,21,22]. The biofilm ensures bacterial survival by making cells less accessible to the host’s defense system and by impairing antibiotic activity. Bacteria present in the biofilm show stronger resistance to antibiotics, whose concentrations must be several times higher to kill microorganisms compared to planktonic cells [23]. The biofilm is permanently attached to the substrate on which it grows, which can be either inanimate matter or body tissues. The biofilm allows bacteria to colonize diverse ecological niches, and in many cases to survive in adverse conditions for individual cells. In the natural environment, over 99% of bacteria occur in the form of a biofilm. The biofilm promotes the survival of bacteria in the host organism, protecting them against immune mechanisms. As a result of biofilm fragmentation and detachment, bacteria can spread in the body, colonizing new places; therefore, biofilm infections are very often chronic and recurrent [24].

Several stages can be distinguished in biofilm development: cell adhesion to the surface, production of microcolonies, biofilm maturation, and detachment of surface biofilm fragments and/or individual planktonic cells [25]. Initial attachment occurs via various proteins, such as Bhp, AtlE, and Fbe, as well as intercellular adhesin. The accumulation stage is characterized by the production of polysaccharide intercellular adhesin (PIA), encoded by *ica*ADBC genes [26,27]. Two major cell-surface-associated proteins, Aap and Embp, have also been shown to contribute to biofilm formation in CoNS. The accumulation protein (Aap) belongs to the Bap family of proteins, and the extracellular matrix-binding protein (Embp) mediates binding of bacteria to fibronectin and is involved in biofilm accumulation [28]. As a result of adhesion to the surface, significant changes in cell metabolism occur, mainly consisting of increased expression of genes encoding the production of extracellular proteins and extracellular secreted polysaccharide substances. The result is cell immobilization in the extracellular matrix, allowing further biofilm formation. 

Although research interest in CoNS has increased in recent years, there are very little data on its prevalence and characteristics in poultry production. Therefore, the purpose of this study was to determine the biofilm-forming potential of coagulase-negative *Staphylococcus* strains isolated from poultry. The frequency of selected genes potentially playing a role in the biofilm formation process was also determined. 

## 2. Materials and Methods

### 2.1. Sample Collection and Identification of Bacterial Strains

The study was conducted on material obtained from broiler chicken farms located in Central-Western Poland between November 2016 and December 2017. During this time, samples from 84 flocks were collected. The samples were taken from the internal organs (heart, liver, spleen, tarsal joints, or bone marrow) of chickens aged 1 day to 6 weeks and showing the following clinical symptoms: increased mortality, dermatitis and cellulitis, lameness and arthritis, decreased weight gain, and/or omphalitis and yolk sac infections. Three to five specimens were taken from the affected organs of each bird. A total of 268 samples were collected from the broilers. The size of the flocks from which the samples were collected ranged from 8000 to 44,000 birds. 

The material (samples of internal organs) was plated on a blood agar medium (Blood LAB-AGAR, Biocorp, Warsaw, Poland) and Chapman selective medium (Mannitol Salt LAB-AGAR, Biocorp, Warsaw, Poland) and incubated under aerobic conditions at 37 °C for 24–48 h, depending on the rate of growth of the bacteria. Single colonies were transferred to blood agar to isolate pure bacterial cultures, and a preliminary bacteriological characterization of the isolated bacteria was performed, involving Gram staining, microscope examination of cell morphology and motility, and determination of the type of hemolysis. Quantitative measurement of the colonies was not performed. Isolated bacteria were stored for further testing at −85 °C in 50% (*v*/*v*) glycerol in brain heart infusion broth (BHI; Sigma-Aldrich, Poznan, Poland). 

All *Staphylococcus* strains were identified by MALDI-TOF MS mass spectrometry using the IVD MALDI Biotyper (Bruker Daltonik, Bremen, Germany), as described by Marek et al. [3].

### 2.2. Biofilm Detection by the Microtiter Plate (MTP) Method

All CoNS isolates were grown overnight at 37 °C on blood agar. Single colonies were inoculated in 3 mL of brain heart infusion broth (BHI; Sigma-Aldrich, Poznan, Poland), incubated for 24 h at 37 °C and then diluted at 1:40 in BHI (2–7 × 10^7^ cfu/mL) using 0.5 MacFarland standard tubes. The microtiter plate test was performed according to Dubravka et al. [29] and Stepanović et al. [30]. For each *Staphylococcus* isolate, 200 μL aliquots of prepared suspension were inoculated into four wells of the 96-well plates (Kartell S.p.A., Noviglio MI, Italy). Each plate included a negative control: four wells with BHI. The plates were incubated at 37 °C for 24 h. Then the contents of each well were removed by aspiration, and the wells were rinsed three times with 250 μL of sterile physiological saline. After the plates were dried, the attached bacteria were fixed for 15 min at room temperature by adding 200 μL of methanol to each well. The plates were stained with 160 μL aqueous solution of crystal violet 0.5% (Crystal Violet, Sigma) for 15 min at room temperature. Then, the plates were rinsed under running water until there was no visible trace of stain. The stain bound to bacteria was dissolved by adding 160 μL of 95% ethanol. The optical density (OD) of each well was measured using an ELISA microplate reader (BIO-RAD, Warsaw, Poland) at 570 nm. Each strain was tested for biofilm production in duplicates and the assay was repeated three times. The following criteria were used for biofilm gradation in clinical isolates. ODc = ODavg of negative control + 3 × standard deviation (SD) of ODs of negative control. 

Strains were interpreted as follows:

(1) non-biofilm producers (OD ≤ ODc);
(2) weak biofilm producers (ODc < OD ≤ 2 × ODc);
(3) moderate biofilm producers (2 × ODc < OD ≤ 4 × ODc);
(4) strong biofilm producers (4 × ODc < OD).

### 2.3. Bacterial DNA Extraction and Detection of Biofilm-Associated Genes

Total DNA was extracted from strains inoculated individually on blood agar and incubated at 37 °C/24 h. The Novabeads Bacterial DNA kit (Novazym, Poznan, Poland) was used for DNA extraction according to the manufacturer’s protocol.

Five biofilm-related genes were analyzed by simplex PCR assays to detect the presence of *ica*AB (*ica* cluster encoding synthesis of polysaccharide intercellular adhesion—PIA), *bap* (encoding biofilm-associated protein), *atl*E (encoding cell surface protein exhibiting vitronectin-binding activity), *fbe* (encoding fibrinogen-binding protein), and *eno* (encoding laminin-binding protein) in all *Staphylococcus* isolates [31,32,33]. The *ica* primers were designed to amplify the *ic*aA and *ica*B genes of the *ica* locus. The nucleotide sequences of the primers are presented in Table 1.

Each 25 μL PCR mixture contained 1.5 U TaqDNA polymerase (EURX, Gdansk, Poland), 1× Standard Taq reaction buffer (10 mM Tris–HCl, 50 mM KCl, 1.5 mM MgCl_2_, pH 8.3), 0.5 μM of each primer, 200 μM dNTPs, and 2 μL of template DNA. Each PCR was performed twice to confirm the results, and each experiment included a PCR-positive control strain and a negative control consisting of the PCR mixture without bacterial DNA.

Reference strains *Staphylococcus epidermidis* ATCC 35984/RP62A (able to form biofilms) and *Staphylococcus epidermidis* ATCC 12,228 (not able to form biofilms) were selected as positive and negative controls in the genetic testing, respectively. Polymerase chain reaction (PCR) conditions for each pair of primers are presented in Table 1. After PCR amplification, 10 μL of PCR product was resolved by 1% (*w*/*v*) agarose gel electrophoresis and visualized by staining with SimplySafe™ (EURX, Gdansk, Poland).

### 2.4. Statistical Analysis

Statistical analysis was performed with Statistica 10.0 software (StatSoft, Krakow, Poland). The statistically significant differences in the frequency of biofilm-related genes in *Staphylococcus* isolates were calculated by Fisher’s exact test, which can be performed online at https://www.langsrud.com/stat/fisher.htm, https://www.graphpad.com/quickcalcs/contingency1, or at https://www.socscistatistics.com/tests/fisher/default2.aspx. Results were considered statistically significant at a *p*-value of <0.05.

## 3. Results

### 3.1. Bacterial Strains

A total of 87 CoNS isolates from broiler chickens were selected in this study. These were strains of *S. epidermidis* (*n* = 17), *S. hominis* (*n* = 4), *S. saprophyticus* (*n* = 19), *S. xylosus* (*n* = 19), *S. haemolyticus* (*n* = 4), *S. sciuri* (*n* = 10), *S. simulans* (*n* = 5), and *S. chromogenes* (*n* = 9), (Table 2).

### 3.2. Biofilm Detection by the Microtiter Plate (MTP) Method

Testing for biofilm production showed that 69 of the 87 CoNS isolated from broiler chickens were biofilm producers, of which 24 isolates (27.6%) were weak biofilm producers, 11 (25.3%) were moderate biofilm producers, and 13 (26.4%) were strong biofilm producers. The ability to form biofilms varied among CoNS species. All isolates of *S. haemolyticus*, *S. sciuri* and *S. simulans* were found to form biofilms. In addition, more than 84% of *S. saprophyticus* and *S. xylosus* isolates were able to form biofilms. In the case of the remaining species, the ability to form a biofilm was observed in a smaller percentage of isolates. The results of the MTP assay of biofilm production by CoNS are presented in Table 3.

### 3.3. Detection of the icaAB, atlE, fbe, bap, and eno Genes

The majority (72; 82.8%) of the isolates were positive for at least one of the five genes tested in different combinations (Table 4), while only 15 isolates (17.2%) were negative for all five genes. The majority of isolates (*n* = 61) were positive for the *eno* gene (70.11%). The presence of the *alt*E gene was demonstrated in 17 *Staphylococcu*s isolates (19.5%). Other genes were found in <10% of the isolates and were found frequently almost exclusively in *S. epidermidis*, *S. xylosus*, *S. simulans,* and *S. saprophyticus*. Detailed data are presented in Table 3 and Table 4.

## 4. Discussion

In the present study, 87 coagulase-negative *Staphylococcus* strains were isolated from broiler chickens. The high number of CoNS isolated in this study could be due to CoNS being abundant in the normal skin and mucosal biota of animals, and some are free-living in the environment [23]. Although normally present, several of these commensal and nonpathogenic staphylococci have been implicated in infections [4]. The predominant CNS species found in this study were *S. xylosus* and *S. saprophyticus*, which are found specifically on the skin and mucous membrane of livestock and birds [39]. In studies published by various authors, the percentage of coagulase-negative *Staphylococcus* species isolated from poultry samples varied significantly. For example, in a study by Boamah et al. [40], the levels of coagulase-negative strains of staphylococci isolated from samples taken from poultry were as follows: *S. sciuri* 42.97%, *S. lentus* 35.94%, *S. xylosus* 4.30%, *S. haemolyticus* 3.91%, *S. saprophyticus* 1.95%, and *S. cohnii* 0.39%. In a report by Simjee et al. [41], 38% of the coagulase-negative *Staphylococcus* spp. was *S. sciuri*, while *S. lentus* and *S. xylosus* constituted 21% and 14%, respectively. In a study published by El-Nagar et al. [42], in Egypt, the CoNS isolates from poultry samples were *S. xylosus* (34.49%), *S. warneri* (17.25%), *S. epidermidis* (10.34%), *S. saprophyticus* (10.34%), *S. simulans* (10.34%), *S. hominis* (10.34%), and *S. capitis* (6.9%). Coagulase-negative *Staphylococcus* species, such as *S. sciuri*, *S. xylosus,* or *S. cohnii,* are considered important poultry pathogens, particularly because they carry genes encoding antimicrobial resistance, biofilm formation, and hemolysin production [40].

Biofilms increase the invasive properties of CoNS and their ability to cause infection [16,43]. The majority (79.3%) of CoNS isolated from broiler chickens in this study were capable of producing a biofilm, and 51.7% were classified as strong or moderate biofilm producers. The data indicated the relatively high prevalence of biofilm-producing CoNS in poultry. Variation was noted in mucus production by strains within individual species. The highest percentage of strains showing strong and moderate biofilm production capacity was observed in the species *S. hominis* (100%), *S. xylosus* (78.9%), *S. sciuri* (60%), *S. simulans* (60%), and *S. saprophyticus* (47.4%). There are many reports in the literature about the special ability of *S. epidermidis* to produce a biofilm [23,44,45]. In our study, among the strains belonging to the species *S. epidermidis*, only 17.6% phenotypically showed strong or moderate biofilm production, whereas as many as 52.9% of these strains were nonadherent. Although biofilms do not appear to contribute to disease severity, they may play a role in the persistence of CoNS in the environment of infection [46]. In a study by Tremblay et al. [46], using a similar test for biofilm production, most of the *Staphylococcus* isolates from cows with mastitis were able to form a biofilm (85%). In contrast, Simojoki et al. [47] observed that most of the tested CoNS (68.7%) were biofilm-negative. This can be attributed to the use of tryptone soy broth (TSB) by Simojoki et al. [47] for bacterial cultivation and brain heart infusion broth (BHI) by Tremblay et al. [46]. The chemical composition of the growth medium (e.g., plant base versus animal organ base) is known to influence the expression of bacterial genes, including exopolysaccharide synthesis genes, and thus biofilm formation by staphylococcal species [48].

The first step in the biofilm formation process is bacterial adhesion to the host extracellular matrix and plasma proteins, mediated by various proteins of the family of microbial surface components-recognizing adhesive matrix molecules (MSCRAMMs). The second step is the growth-dependent accumulation of bacteria in multilayered cell clusters (intercellular adhesion), where genes involved in biofilm formation play a role [26,35]. However, there are no literature data concerning the presence of MSCRAMM genes in poultry CoNS isolates. In our study, the majority of isolates were positive for the *eno* gene (70.1%), which was also the dominant gene (75% of isolates) detected by Simojoki et al. [47]. The *eno* gene was found to be widely distributed, irrespective of species and biofilm-forming ability. It is therefore likely that *eno* is not a biofilm-specific gene. The percentage of positive results for the intercellular adhesion gene (*ica*AB), the key factor in the aggregation stage, was 6.9%, which is similar to the rates found by Simojoki et al. [47] and Srednik et al. [49] in staphylococci isolates from cattle with mastitis. In our study, the highest percentage of strains in which the presence of the *ica*AB gene was detected belonged to the species *S. xylosus*. The biofilm-associated protein (bap) is vital for the primary attachment of bacteria and biofilm formation [35]. This gene was identified in a small proportion of *S. aureus* isolates from bovine mastitis. Bap orthologue genes have also been found in other staphylococcal species, including *S. epidermidis*, *S. chromogenes*, *S. xylosus*, *S. simulans,* and *S. hyicus* [35]. In our study, the presence of the *bap* gene was detected in only a few isolates (*n* = 5), and the highest percentage of strains in which it was detected also belonged to the species *S. xylosus*, which confirms the observation that *bap* is rare outside of *S. xylosus* isolates [46].

A recent study demonstrated that the primary attachment of *S. epidermidis* to a polystyrene surface is associated with a cell surface protein exhibiting vitronectin-binding activity. This protein is encoded by the chromosomal *atl*E gene and exhibits high similarity to the major autolysin of *S. aureus* [32]. The presence of the autolysin (*atl*E) gene was demonstrated in as many as 19.5% of the tested isolates belonging to the species *S. epidermidis*, *S. saprophyticus*, *S. chromogenes*, and *S. haemolyticus.* However, the fibrinogen-binding protein gene (*fbe*) was found in only 8% of the tested isolates belonging to the species *S. epidermidis*, *S. xylosus*, and *S. simulans*. In our study, the *fbe* and *atl*E genes were present in five strains of *S. epidermidis*, which constituted 29.4% of isolates belonging to this species. This result differs significantly from that obtained by Lianhua et al. [50], who detected these genes in 94.6% of human clinical isolates of *S. epidermidis*.

Notably, there are some limitations in detecting a selected MSCRAMMs genes and associating them with the biofilm phenotype. Our results showed that not all *ica*AB gene-carrying isolates had the capacity to produce biofilms and that the *ica*AB gene was not found in most biofilm-producing strains. It was reported that the correlation between *ica* carriage and the biofilm-forming ability of *Staphylococcus* bacteria is unpredictable because the expression of biofilm-depending genes and the adhesion on surfaces are complex processes of gene regulation dependent on several factors as well as nutrients, pH value, and surface characteristics [51].

## 5. Conclusions

The growing role of coagulase-negative staphylococci in animal infections necessitates their accurate identification, which in turn will enable precise determination of the pathogenic role of particular species. The results of the study indicate that poultry can be a source of coagulase-negative staphylococci capable of forming biofilms, which is considered a clinically important virulence factor of these bacteria. The study showed that almost all CoNS strains from broilers are able to form a biofilm, and over half of the isolates were strong or moderate biofilm producers. This indicates that biofilm formation in CoNS species is unlikely to occur due to a single component and/or process. Furthermore, the ability of these species to form a biofilm limits treatment possibilities and thus may increase the morbidity and mortality rate in poultry. The *eno* gene was the only MSCRAMM gene commonly detected in CoNS in poultry isolates. Biofilm production by CoNS may occur irrespective of the presence of *ica*AB genes.

Demonstration of genes that play a role in bacterial biofilm formation may serve as a genetic basis to distinguish between symbiotic and potentially invasive coagulase-negative staphylococcal strains.

## Figures and Tables

**Table 1:** Nucleotide sequences and sizes of PCR products of amplified genes.

Gene *	Oligonucleotide Sequence (5′-3′) **	Amplicon Size (bp)	PCR Conditions	References
*ica*AB	TTATCAATGCCGCAGTTGTC GTTTAACGCGAGTGCGCTAT	546	94 °C, 5 min; 30 cycles of 94 °C for 30 s, 55 °C, 1 min, 72 °C, 1 min, final extension 72 °C, 5 min.	[34]
*atl*E	CAACTGCTCAACCGAGAACA TTTGTAGATGTTGTGCCCCA	682	94 °C, 2 min; 30 cycles of 94 °C for 1 min, 55 °C, 1 min, 72 °C, 2 min, final extension 72 °C, 5 min.	[35]
*fbe*	TAAACACCGACGATAATAACCAAA GGTCTAGCCTTATTTTCATATTCA	495	94 °C, 3 min; 30 cycles of: 94 °C, 1 min; 62 °C, 1 min; 72 °C, 1 min; final extension 72 °C, 5 min	[36]
*bap*	CCCTATATCGAAGGTGTAGAATTG GCTGTTGAAGTTAATACTGTACCTGC	971	94 °C, 2 min; 40 cycles of 94 °C, 30 s, 55 °C, 30 s, 72 °C, 75 s; final extension 72 °C, 5 min.	[37]
*eno*	ACGTGCAGCAGCTGACT CAACAGCATYCTTCAGTACCTTC	302	94 °C, 5 min; 35 cycles of 94 °C, 1 min, 56 °C, 1 min, 72 °C, 1 min, final extension 72 °C, 10 min.	[38]

* The sets of primers were synthesized by Genomed S.A, Poland; ** The concentration of primers was 0.04 μmol; F—forward primer; R—reverse primer.

**Table 2:** The distribution of isolated *Staphylococcus* species in various tissues and organs of broilers.

CoNS Source n (%)	*S. epidermidis* 17 (19.6)	*S. hominis* 4 (4.6)	*S. saprophyticus* 19 (21.8) ^A^	*S. xylosus* 19 (21.8) ^A^	*S. haemolyticus* 4 (4.6) *^BC^	*S. sciuri* 10 (11.5)	*S. simulans* 5 (5.8) *^BC^	*S. chromogenes* 9 (10.3)	Total Strains (%) 87 (100)
heart	5	-	5	2	3	1	4	2	22 (25.3)
liver	2	-	-	5	-	3	-	3	13 (14.9)
spleen	4	1	4	-	1	3	1	4	18 (20.7)
tarsal joints	6	3	8	9	-	1	-	-	27 (31.0)
bone marrow	-	-	2	3	-	2	-	-	7 (8.1)

Statistically significant difference (*p* < 0.005) between * the analyzed species and *S. epidermidis,* ^A^ the analyzed species and *S. hominis*, ^B^ the analyzed species and *S. saprophyticus,* and ^c^ between the analyzed species and *S. xylosus*. CoNS, coagulase-negative staphylococci

**Table 3:** Biofilm formation on polystyrene 96-well plates and biofilm-associated genes among CoNS species.

Item	*S. epidermidis*	*S. hominis*	*S. saprophyticus*	*S. xylosus*	*S. haemolyticus*	*S. sciuri*	*S. simulans*	*S. chromogenes*	Total Strains (%)
**n** **(%)**	17 (19.6)	4 (4.6)	19 (21.8)	19 (21.8)	4 (4.6)	10 (11.5)	5 (5.8)	9 (10.3)	87 (100)
**Biofilm**									
**Nonadherent**	9		3	3				3	18 (20.7)
**Weak**	5		7	1	1	4	2	4	24 (27.6)
**Moderate**	2	1	3	6	3	5		2	22 (25.3)
**Strong**	1	3	6	9		1	3		23 (26.4)
**Biofilm genes**									
**icaAB**			1	4			1		6 (6.9)
**bap**			1	3				1	5 (5.7)
**fbe**	5			1			1		7 (8.0)
**atlE**	5		5		1			6	17 (19.5)
**eno**	11	4	18	16	3	5	3	1	61 (70.1)

**Table 4:** Biofilm-associated gene patterns.

Biofilm-Associated Gene Combinations	Number of Isolates (%)
*eno*	40 (46) *
*atl*E	8 (9.2)
*fbe*	2 (2.3)
*bap*	1 (1.2)
*ica*AB*-eno*	6 (6.9)
*atl*E*-eno*	6 (6.9)
*fbe-eno*	3 (3.4)
*bap-eno*	3 (3.4)
*atl*E-*eno-bap*	1(1.2)
*atl*E*-fbe-eno*	2 (2.3)

* Statistically significant difference (*p* < 0.001) between the *eno* gene and the other studied genes.

## Data Availability

Data is contained within the article.
